# There may be a link between intrahepatic cholestasis of pregnancy and familial combined hyperlipidaemia: a case report

**DOI:** 10.4076/1757-1626-2-8679

**Published:** 2009-09-17

**Authors:** Tosin Ajala, Junaid Rafi, Richard Wray, Mark William Whitehead, Jamal Zaidi

**Affiliations:** 1 Department Obstetrics and Gynaecology, Basingstoke & North Hampshire NHS Trust, Aldermaston Road, Basingstoke, RG24 9NA, UK; 2 Department of Cardiology, Conquest Hospital, The Ridge St. Leonards on Sea, East Sussex, TN37 7RD, UK; 3 Department of Gastroenterology, Conquest Hospital, The Ridge St. Leonards on Sea, East Sussex, TN37 7RD, UK; 4 Department Obstetrics and Gynaecology, Conquest Hospital, The Ridge St. Leonards on Sea, East Sussex, TN37 7RD, UK

## Abstract

A 26-year-old gravida 3 para 1+1 was referred for antenatal care. In her last pregnancy she had a early spontaneous preterm delivery at 32 weeks and 2 days complicated by intra hepatic cholestasis of pregnancy. She had a strong family history of ischemic heart and combined hyperlipidaemia. In view of her past obstetric history a baseline liver function test and fasting bile acid assay was carried out. Upto 21 week her Bile acids were normal but at 22 weeks her fasting bile acid assay increased to the upper limit of normal (9 µmol/L).

Ursodeoxycholic acid was started from 28 weeks gestation on a dosage of 500 mg b.i.d., which was subsequently increased to 500 mg t.d.s. at 32 weeks.

At 34 weeks she gave a history of occasional right upper quadrant abdominal pain and her biochemistry revealed raised serum aspartate transaminase ,alanine transaminase, fasting serum triglyceride and cholesterol levels 58 IU,79 IU/L,18.37 mmol/L and 25.7 mmol/L respectively. The triglyceride level was too high to calculate the low density lipoprotein cholesterol. A diagnosis of severe intrahepatic cholestasis of pregnancy in a patient with background familial combined hyperlipidaemia was made. Ultrasound abdomen and cardiotocography was normal. She had normal delivery. In cases of early onset cholestasis of pregnancy we suggest that lipid profiles are checked in these patients to rule out hyperlipidaemia and its attendant short term and long-term risks. More research will be required to ascertain if there is a link between these 2 disorders.

## Introduction

We report a case of severe early onset reoccurring intrahepatic cholestasis of pregnancy (ICP) in a patient with exacerbation of familial combined hyperlipidaemia (Fredrickson/WHO type V). To our knowledge this is the first described case.

Cholestasis of pregnancy is a rare disorder of unknown aetiology occurring in 1/1000-10,000 pregnancies [1]. It usually presents in the 3^rd^ trimester of pregnancy and rarely in the second trimester before 25 weeks gestation. It characteristically presents as pruritus initially of the palms and soles mainly at night, progressively spreading to other parts of the body and increasingly becoming more persistent. The prevalence of jaundice varies from 17-75% [1]-[3]. While perinatal mortality can be as high as 20%, maternal monitoring of fetal movement and electronic fetal monitoring have been shown to be unreliable in preventing foetal loss [4].

## Case presentation

A 26-year-old (Caucasian British, gravida 3 para 1+1) was referred to the Conquest Hospital, East Sussex at 8 weeks gestation for antenatal care.

She had suffered an early miscarriage at 9 weeks gestation with her first pregnancy and subsequently a spontaneous preterm delivery at 32 weeks and 2 days with her following pregnancy. That pregnancy had been complicated by intrahepatic cholestasis of pregnancy (ICP).

She had a strong family history of ischemic heart disease. On her maternal side, her grandfather had died of myocardial infarction (MI) at the age of 61 years and her uncle had an MI at the age of 45 years. On her paternal side of the family, her uncle aged 41 years, had suffered an acute coronary event. Her father aged 61 years, has combined hyperlipidaemia. She is a non-smoker.

At her booking visit at 10 weeks gestation clinical examination was unremarkable and her body mass index was normal. In view of her past obstetric history a baseline liver function test (LFT) and fasting bile acid assay (BA) was carried out. Her alanine transaminase (ALT) was slightly elevated 42 IU/L (3-31) otherwise her LFT and fasting BA assay were within normal limits, bile acid =3 µmol/L (Normal limits 0-10). At her next appointment (16 weeks gestation), she admitted to itching of her palms and soles mainly at night. A repeat LFT was all within normal limits as was the fasting BA, 6 µmol/L. She was again seen 2 weeks later in the antenatal clinic at 18 weeks gestation, her itching had worsened, and otherwise routine antenatal checks were normal. Her fasting BA assay remained normal 4 µmol/L, she was started on piriton tablets and calamine lotion and given a further appointment at 20 weeks at which her symptoms persisted and the fasting BA remained normal (6 µmol/L), as did her management.

Subsequent fetal anomaly scan carried out at 21 weeks gestation was normal.

At 22 weeks her fasting BA assay increased to the upper limit of normal (9 µmol/L), her LFT remained normal and in view of her persistent itching, it was recommended that she started ursodeoxycholic acid (UDCA), but this was declined. She continued on piriton tablets 4 mg 8 hourly. A follow-up appointment was arranged for 24 weeks, at this appointment she said that all was well, her latest fasting BA assay was 5 µmol/L.

However she admitted feeling unwell, experiencing some uterine contractions and reduced fetal movements at 26 weeks gestation. Routine examinations were normal. Ultrasound fetal biometric measurements were consistent with good growth and cardiotocography (CTG) tracing was reactive with evidence of fetal activity. LFT, urea and electrolytes were all within normal range. For the first time in this pregnancy her fasting BA assay was slightly above normal range (12µ mol/L). In view of her past obstetric history, she was given 2 doses of 12 mg of betamethasone intramuscular injections 24 hours apart. At 28 weeks gestation she admitted to worsen of her itching mainly at night, her fasting BA assay was 13 µmol/L and her LFT remained normal. She was commenced on UDCA 500 mg twice daily orally, vitamin K 10 mg daily and aqueous menthol cream at night for relief of itching.

We decided to see her weekly for clinical review and monitoring of her biochemistry. Her symptoms remained unchanged over the following 3 weeks as did her biochemistry notably her fasting BA assay 7, 1 and 7 µmol/L at 29, 30 and 31 weeks respectively.

Fetal growth ultrasound scan and CTG monitoring were reassuring.

At 32 weeks gestation, she complained of worsening pruritis, she noticed that her urine had become darker but denied change in the colour of her faeces. Her serum BA shot up to 161 µmol/L, while her LFT remained normal. The decision was made to increase the UDCA to 500 mg t.d.s.

At 34 weeks she gave a history of occasional right upper quadrant abdominal pain associated with vomiting described as "soap-like". On examination she was anicteric, normotensive with vague upper right abdominal tenderness. Her biochemistry revealed raised serum aspartate transaminase (AST) and ALT 58 and 79IU/L respectively. The blood sample was grossly lipaemic; fasting serum triglyceride and cholesterol levels were significantly raised 18.37 mmol/L and 25.7 mmol/L respectively. The triglyceride level was too high to calculate the low density lipoprotein cholesterol (LDLC). Her serum amylase level was normal 17IU/L as was her thyroid function test. Her fasting BA assay was raised; 134 µmol/L. Upper abdominal ultrasounds scan revealed a normal liver and pancreas with evidence of multiple gall bladder calculi. Fetal wellbeing was confirmed by reactive CTG and satisfactory growth on ultrasound scan.

A diagnosis of severe ICP in a patient with background familial combined hyperlipidaemia was made. We excluded acute fatty liver of pregnancy and closely observed her for the onset of superimposed acute pancreatitis in the presence of the hypertriglyceridaemia.

The patient was placed on reduced fat diet to less than 10%, increased fluid intake (3 Litres /24 hours), daily serum amylase and lipid monitoring. We planned to induce labour at 34 weeks and 3 days.

She presented at 34 weeks and 2 days in established labour and following a 7-hour labour had a spontaneous vaginal delivery of a live 2550 g male baby. Apgar scores were 5 @ 1 minute and 8 @5 minutes.

Her lipid profile was assessed on day 3, 5, 9 and 6 weeks postpartum (Table 1).

**Table 1:** Postnatal lipid profile

Post-op (day)/ weeks	Cholesterol (mmol/L)	Triglyceride (mmol/L)
0	22.30	14.97
3	19.0	15.78
5	17.30	15.28
9	13.40	13.89
6 weeks	8.10	3.97

## Discussion

Physiological hyperlipidaemia of pregnancy consist primarily of an increase in triglycerides, with a lesser degree of a rise in cholesterol and phospholipids in pregnancy [5]. This physiological change is exacerbated in cases with underlying familial hyperlipidaemia, with the attendant risk of cholesterol gall stone formation and acute pancreatitis secondary to the hypertriglyceridaemia.

Our patient presented with a known history of severe ICP and a strong family history of familial combined hyperlipidaemia. She had never been diagnosed with hyperlipidaemia prior to this pregnancy. This is probably explained by the absence of clinical features, such as tuberoeruptive xanthoma or hepatomegaly. Her previous preterm delivery at 32 weeks, a known complication of cholestasis of pregnancy [6], may have predated the excessive increase in serum level of triglycerides as to induce the lipaemic serum which aroused suspicion in this case.

While the aetiology of cholestasis of pregnancy remains unknown, studies of the pathogenesis indicate abnormalities in the metabolism of sex hormones and BA.

Clinical studies support an aetiological role of estrogens. These include the relationship between onset of disease and high levels of oestrogen in the third trimester, higher incidence of cholestasis of pregnancy in twin pregnancies and similar symptoms in postnatal patients that use the combined oral contraceptive pills for contraception [7]. Progesterone and BA metabolism in patients with cholestasis of pregnancy has been shown to differ from that of healthy patients. While the overall progesterone synthesis remains unchanged from that in normal pregnancies, patients with ICP have been shown to have a higher proportion of mono and disulfated metabolites of serum progesterone. Glucuronidated metabolites are however similar to that of healthy pregnant patients. This has led to the suggestion of a selective defect in the biliary excretion of sulphated progesterone metabolites into bile [8].

Cholesterol is essential in the production and integrity of the cell membrane; it is also the precursor for the synthesis of steroid hormones and BA. Exported cholesterol from the liver and intestines exceed peripheral catabolism, except during growth or tissue repair. The excess cholesterol is returned to the liver (reverse cholesterol transport process) and eliminated in bile as BAs and faecal sterols. This process of BA synthesis is one of the predominant means of excretion of excess cholesterol [9]-[10]. Up regulation of BA synthesis by lipoprotein cholesterol through increased cholesterol 7-alpha hydroxylase (the rate determining step for conversion of cholesterol to BAs) has been shown in in-vitro animal studies [11].

The role that the underlying familial combined hyperlipidaemia played in the severity of her ICP remains unclear. We suspect that while she probably had a genetic predisposition to ICP [7], her excessive circulating cholesterol could have been an aggravating factor.

**Figure 1:**
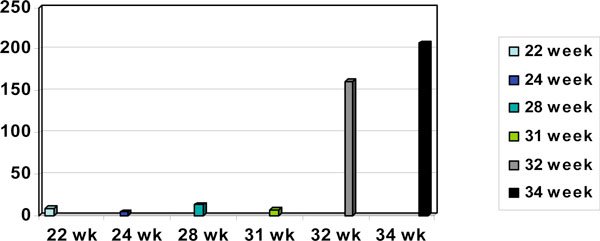
**Trend of bile acids (umol/L) levels at different gestations**. Ursodeoxycholic acid 500 mg bd from 28 weeks gestation, increased to tds from 32 weeks gestation.

UDCA, a hydrophilic BA was started from 28 weeks gestation on a dosage of 500 mg bd, which was subsequently increased to 500 mg tds at 32 weeks following a sudden steep rise in serum BA level to 161 µmol/L and finally peaked at 207 µmol/L at 34 weeks (Figure 1). While there have been some encouraging reports from some small studies, mainly in the improvement of BA levels and LFT, meta-analysis of the use of UDCA have failed to show significant benefits in the use of this medication for the persistent alleviation of pruritus [12].

In the presence of the patient's underlying hyperlipidaemia, early management with low fat diet would have improved her lipid state [13] reducing the attendant risk of acute intrapartum pancreatitis that is associated with a maternal mortality as high as 20%, which was a real concern in our case [14]. We also suggest that improved lipid profile may have improved our control of the ICP.

## Conclusion

In cases of early onset cholestasis of pregnancy we suggest that lipid profiles are checked in these patients to rule out hyperlipidaemia and its attendant short term and long-term risks. More research will be required to ascertain if there is a link between these 2 disorders.

## Abbreviations

ALT: alanine transaminase; AST: aspartate transaminase; BA: Bile acids; CTG: cardiotocography; ICP: intrahepatic cholestasis of pregnancy; LDLC: low density lipoprotein cholesterol; LFT: liver function test; MI: myocardial infarction; UDCA: ursodeoxycholic acid.

## Consent

Written informed consent was obtained from the patient for publication of this case report and any accompanying images. A copy of the written consent is available for review by the Editor-in-Chief of this journal

## Competing interests

The authors declare that they have no competing interests

## Authors' contributions

TA and JR was the major contributor in writing. RW, MW and JZ were involved in management. They also analyzed and interpreted the patient data revised it critically for important clinical and educational content.

## References

[B1] Reyes H Review: Intrahepatic cholestasis. A puzzling disorder of pregnancy J Gastroenterol Hepatol 1997 12 211 216 10.1111/j.1440-1746.1997.tb00410.x 9142637

[B2] Brites D Rodrigues CM Oliveira N Cardoso M Graça LM Correction of maternal serum BA profile during ursodeoxycholic acid therapy in cholestasis of pregnancy J Hepatol 1998 28 91 98 10.1016/S0168-8278(98)80207-9 9537870

[B3] Borum ML Hepatobiliary diseases in women Med Clin North Am 1998 82 51 75 10.1016/S0025-7125(05)70594-0 9457151

[B4] Rioseco AJ Ivankovic MB Manzur A Hamed F Kato SR Parer JT Germain AM Intrahepatic cholestasis of pregnancy: A retrospective case-control study of perinatal outcome Am J Obstet Gynecol 1994 170 890 895 8141222 10.1016/s0002-9378(94)70304-3

[B5] Warth MR Arky RA Knopp RH Lipid metabolism in pregnancy. II. Altered lipid composition in the intermediate, very low, low and high-density lipoprotein fractions J Clin End & Metab 1975 41 649 655 170295 10.1210/jcem-41-4-649

[B6] Reid R Ivey KJ Rencoret RH Storey B Fetal complications of obstetrics cholestasis Br Med J 1976 1 807 872 10.1136/bmj.1.6014.870 1083274 PMC1639586

[B7] Germain AM Nien JK Mena B Carvajal JA IRIS—International Registry of Intra-hepatic Cholestasis of Pregnancy-Related Stillbirth Front in Fetal Health 2001 2 1 6

[B8] Reyes H Sjovall J BAs and progesterone metabolites in intrahepatic cholestasis of pregnancy Ann Med 2000 32 94 106 10.3109/07853890009011758 10766400

[B9] Bjorkhem I Danielsson HSjovall J In Sterols and Bile Acids 1985 12 Elsevier 231 278 10.1016/S0167-7306(08)60685-7

[B10] Carey MC Duane WC Arias IMBoyer JLFausto NJakoby WBSchachter DAShafritz DA The liver. Biology and Pathobiology 1994 3 Raven 719 767

[B11] Post SM Twisk J van der Fits L de Wit EC Hoekman MF Mager WH Princen HM Lipoprotein cholesterol uptake mediates up-regulation of BA synthesis by increasing cholesterol 7 alpha-hydroxlase but not sterol 27-hydroxylase gene expression in cultured rat hepatocytes Biochem J 1999 341 339 346 10.1042/0264-6021:3410339 10393091 PMC1220365

[B12] Burrows RF Clavisi O Burrows E Interventions for treating cholestasis in pregnancy Cochrane Database Syst Rev 2001 (4) CD000493 11687082 10.1002/14651858.CD000493

[B13] Glueck CJ Christopher C Mishkel MA Tsang RC Mellies MJ Pancreatitis, familial hypertriglyceridaemia and pregnancy Am J Obstet Gynecol 1980 136 755 7355961 10.1016/0002-9378(80)90452-4

[B14] Montgomery WH Miller FG Pancreatitis and pregnancy Obstet Gynecol 1986 35 1054 5438159

